# Clobetasol promotes remyelination in a mouse model of neuromyelitis optica

**DOI:** 10.1186/s40478-016-0309-4

**Published:** 2016-04-26

**Authors:** Xiaoming Yao, Tao Su, A. S. Verkman

**Affiliations:** Departments of Medicine and Physiology, University of California San Francisco, 1246 Health Sciences East Tower, San Francisco, CA 94143-0521 USA

**Keywords:** NMO, Clobetasol, Demyelination, Oligodendrocyte, Mouse models

## Abstract

Neuromyelitis optica (NMO) is an inflammatory demyelinating disease of the central nervous system that can produce marked neurological deficit. Current NMO therapies include immunosuppressants, plasma exchange and B-cell depletion. Here, we evaluated 14 potential remyelinating drugs emerging from prior small molecule screens done to identify drugs for repurposing in multiple sclerosis and other demyelinating neurological diseases. Compounds were initially evaluated in oligodendrocyte precursor cell (OPC) and cerebellar slice cultures, and then in a mouse model of NMO produced by intracerebral injection of anti-AQP4 autoantibody (AQP4-IgG) and human complement characterized by demyelination with minimal axonal damage. The FDA-approved drug clobetasol promoted differentiation in OPC cultures and remyelination in cerebellar slice cultures and in mice. Intraperitoneal administration of 2 mg/kg/day clobetasol reduced myelin loss by ~60 %, even when clobetasol was administered after demyelination occurred. Clobetasol increased the number of mature oligodendrocytes within lesions without significantly altering initial astrocyte damage or inflammation. These results provide proof-of-concept for the potential utility of a remyelinating approach in the treatment of NMO.

## Introduction

Neuromyelitis optica (NMO) is a neuroinflammatory demyelinating disease that affects spinal cord and optic nerve, and to a lesser extent brain. Most NMO patients are seropositive for IgG1-class autoantibodies against astrocyte water channel aquaporin-4 (AQP4). It is thought that the anti-AQP4 autoantibodies (called AQP4-IgG) are pathogenic in NMO by a mechanism involving complement- and cell-dependent astrocyte damage and an inflammatory response, which leads to oligodendrocyte injury, demyelination and neurological deficit [6, 29, 36]. Current NMO therapeutics include immunosuppressants, plasma exchange and B-cell depletion therapy [42]. Alternative targets under consideration for NMO therapy include the AQP4-IgG antibody and its binding to AQP4 [40], complement and complement inhibitory proteins [32, 33] and various immune cells including plasma cells and granulocytes [7, 11, 16, 28].

Here, we evaluated remyelination as a potential therapeutic approach in NMO, with the goal of reducing axonal degeneration and neuronal loss following demyelination associated with disease exacerbations, which could reduce cumulative neurological deficit. Though the subject of remyelination therapeutics is under active investigation for multiple sclerosis [12, 17, 26], remyelination has received little attention in NMO, perhaps in part because of theoretical challenges in effecting remyelination in NMO, because: *(i)* primary astrocyte damage in NMO could interfere with oligodendrocyte-astrocyte interactions that might be important in oligodendrocyte functions [5, 19, 45]; *(ii)* blood–brain barrier disruption in NMO could inhibit oligodendrocyte migration along microvessels [41]; and *(iii)* the inflammatory environment in active NMO lesions could inhibit remyelination and produce irreversible axonal injury. However, limited analysis of early pathology in NMO suggests similar axonal preservation in NMO and multiple sclerosis [3], which would support the evaluation of remyelinating therapeutics in NMO.

Notwithstanding these challenges, here we investigated the potential efficacy of small molecule remyelinating compounds in NMO. For in vivo studies we modified an established, passive-transfer mouse model of NMO in which intracerebral administration of AQP4-IgG and human complement by stereotaxic infusion produces characteristic NMO pathology with loss of AQP4 and GFAP, complement deposition, inflammation and demyelination, but with minimal axonal damage. We evaluated 14 potential remyelination drugs, as listed in Table 1, based on our review of the literature and selection of those drugs that have a mechanism consistent with use in NMO and for which the data are most clear-cut. All compound have been identified in in vitro drug screens of oligodendrocyte precursor cell (OPC) maturation or function. One compound, the approved drug clobetasol, promoted oligodendrocyte maturation in the primary OPC cultures, and remyelination in AQP4-IgG treated cerebellar slice cultures and mice, providing proof of concept for remyelinating therapy in NMO.

**Table 1:** Compounds with reported remyelinating activity

Compound	Model systems	Proposed mechanisms	References
Benztropine	EAE, cuprizone mouse model	Muscarinic agonist	[8]
CDP-choline	EAE, cuprizone mouse model	Protein kinase C-mediated OPC proliferation	[37]
Clemastine	Lysolecithin mouse model	Antihistamine, anticholinergic	[20]
Clobetasol	Lysolecithin model, EAE	Glucocorticoid receptor signaling, Hedgehog signaling, OPC differentiation	[10, 24, 27, 34, 44]
Enprofylline	Kainic acid-induced spinal cord injury ex vivo	Adenosine receptor antagonist	[21]
Fasudil	OPC culture	Rho-kinase inhibitor, vasodilator	[2]
GC-1	OPC culture/P7 mouse model of myelination	Thyroid receptor agonist, OPC differentiation	[4]
Indazole	EAE	Estrogen receptor beta agonist	[22]
Miconazole	Lysolecithin model, EAE	ERK1/2 activator, OPC differentiation	[14, 24]
Olesoxime	Lysolecithin mouse model, cuprizone mouse model	Mitochondrial pore modulator	[18]
Quercetin	EAE	γ-secretase inhibition interfering with canonical Notch signaling	[15]
Quetiapine	EAE, cuprizone model, cerebral ischemia	Free radical scavenging, neurotrophic factor stimulation	[51]
Retinoic acid	Lysolecithin model, ethidium bromide model	Retinoid X receptor γ agonist	[13]
Y-27632	Lysolecithin in cerebellar slice cultures	Rho-kinase inhibitor	[31]

## Materials and methods

### Mice

Experiments were done on male wild-type mice on a CD1 genetic background of age 10–12 weeks. Mice were maintained in air-filtered cages and fed normal mouse chow in the UCSF animal facility. All animal procedures were approved by the UCSF Institutional Animal Care and Use Committee.

### Compounds and NMO antibody

Purified human monoclonal recombinant AQP4-IgG rAb-53 (AQP4-IgG) was provided by Dr. Jeffrey Bennett (Univ. Colorado Denver) as described [45]. Control human IgG (control-IgG) was purchased from Pierce Biotechnology (Rockford, IL, USA). Human complement (HC) was purchased from Innovative Research (Novil, MI, USA). Test drugs included clobetasol, miconazole, benztropine, clemastine, fumarate, retinoic acid and citicolone (Sigma-Aldrich, St. Louis, MO, USA), enprofylline, olesoxime and quetiapine fumarate (Santa Cruz Biotechnology, Dallas, TX, USA), GC-1 and quercetin (Tocris Bioscience, Bristol, UK), fasudil (Tszchem, Lexington, MA, USA), and Y-27632 (BD Biosciences, San Jose, CA, USA); Triiodothyronine (T3, Calbiochem, Billerica, MA, USA) was used as positive control. Drugs were dissolved in 2.5 % DMSO + 2 % solutole in PBS. Unless otherwise specified all other chemicals and media were purchased from Sigma-Aldrich.

### Primary culture of mouse oligodendrocyte precursor cells (OPCs)

OPC cultures from mice were generated as described [9] with modifications. Briefly, whole mouse brain was harvested from ice-anesthetized postnatal day 7 pups and brain cortexes were isolated and placed in a pre-chilled Petri-dish containing Hank’s balanced salt solution (HBSS, pH 7.2; Invitrogen, Camarillo, CA, USA) without Ca^2+^ and Mg^2+^. After removal of the meninges, cortexes were diced and digested for 20 min at 37 °C in Ca^2+^ and Mg^2+^-free HBSS containing 20 units/ml papain, 5 mM L-cysteine and 20 units/ml DNase I. The enzyme reaction was stopped by adding Dulbecco’s Modified Eagle Medium (DMEM) containing 10 % Fetal Bovine Serum (FBS) and trypsin inhibitors. The brain tissue was then passed 5 times through an 18-gauge needle and centrifuged at 1200 g for 5 min. The tissue pellet was resuspended in 6 ml of DMEM containing 10 % FBS and incubated at 34 °C for 1 h with gentle shaking. The tissue suspension was passed through a 70-μm nylon strainer and then added onto 15 % Percoll in DMEM and centrifuged at 1200 g for 15 min to remove myelin and large debris. OPCs were enriched using anti-O4 MicroBeads (Miltenyi Biotec, San Diego, CA, USA) and magnetic cell sorting (MACS). The cell pellet was suspended at ~2 × 10^7^ cells in 180 μl BSS containing 25 μl of anti-O4-MicroBeads. After incubation at 4 °C for 15 min the cell suspension was passed 3 times through MACS LS columns. Bound cells were eluted by OPC culture medium consisting of NeuroBasal medium (Gibco, Grand Island, NY, USA) containing 1 % FBS, B27-without vitamin A (Invitrogen, Camarillo, CA), non-essential amino acids, L-glutamine and PDGF-AA (25 ng/ml; ProSpec-Tany TechnoGene, Rehovot, Israel). The OPC yield with this method was 5 × 10^5^ cells per brain, and the purity of oligodendrocytes was greater than 95 %. Cells were cultured on poly-D-lysine coated 75 cm^2^ tissue culture flasks and passed three times for experiments.

For compound testing, OPC cultures at passage three were sub-cultured (25,000 cells/well) on laminin-coated Falcon 8-well-culture slides (Corning, NY, USA). After 24 h, test compounds were added in OPC culture medium with reduced PDGF (5 ng/ml). Cells were cultured for 6 days, with media (with compound) changed every 2 days. Cells were then fixed with PBS containing 4 % PFA with 0.05 % Triton X-100 for immunostaining for MBP and Olig2. The numbers of Olig2- and MBP-positive cells were counted at 400x with six fields in each of three different wells counted per compound. In addition, MBP-positive cells were scored for maturation level at a scale grade of 1 to 3, as described [38, 47]; grade 1 - simple primary branches; grade 2 - medium-size secondary branches, grade 3 - many tertiary branches.

### Organotypic cerebellar slice cultures and ex vivo NMO model

Cerebellar slice cultures were prepared using an interface-culture method as described [24, 39] with modification. Postnatal day 7 mouse pups in a CD1 genetic background were decapitated and the whole cerebellum was rapidly removed, placed in ice-cold Hank’s balanced salt solution (HBSS, pH 7.2; Invitrogen) and embedded in 2 % low-melting agarose. Parasagittal slices of 300-μm thickness were cut using a vibrating microtome (VT-1000S; Leica, Wetzlar, Germany). Individual slices were placed on transparent, non-coated membrane inserts (Millipore, Millicell-CM 0.4-μm pores, 30-mm diameter) in six-well (35-mm diameter) plates containing 1 mL culture medium (50 % MEM, 25 % HBSS, 25 % horse serum, 1 % penicillin–streptomycin, 0.65 % glucose and 25 mM HEPES), with a thin film of culture medium covering slices. Slices were cultured in 5 % CO_2_ at 37 °C for 7 days and medium was changed every 2 days. AQP4-IgG (5 μg/ml) or control-IgG (5 μg/ml) with 5 % human complement were added on day 7 to the culture medium for 20 h. Drugs were then given after removal of AQP4-IgG or control-IgG. After 6 day of treatment slices were fixed in 4 % PFA for whole-mount immunostaining.

### Mouse model of NMO

A mouse model of NMO was created by intracerebral injection of AQP4-IgG and human complement as described [35, 36] with modification to obtain demyelination with minimal axonal damage. A brain NMO model was used here for technical reasons, as injection can be accomplished with minimal trauma, and pathology is highly reproducible. Mice were anesthetized with ketamine (100 mg/kg) and xylazine (10 mg/kg intraperitoneal) and mounted on a stereotaxic frame. A midline scalp incision was made and a burr hole of diameter 1 mm was drilled on each side of skull 0.5 mm anterior and 2 mm lateral to the bregma. A glass pipette with 40-μm tip made on a micropipette puller (Model P-97, Sutter Instrument Co., Novato, CA, USA) was inserted 3-mm deep to infuse AQP4-IgG (or control-IgG) and 1 μl human complement (HC) in a total volume of 3 μl over 10 min by pressure injection (PMI-100, DAGAN Corporation, MN, USA) at 10 psi. After injection, the glass pipette remained in place for 10 min before slow withdrawal (over 5 min) to prevent leaking. For drug testing, mice were treated with test compound (in 2.5 % DMSO + 2 % solutole with PBS) or vehicle starting on day 1 and sacrificed on day 9. Some mice were treated with drug or vehicle starting on day 4 and sacrificed on day 9. At the time of sacrifice mice were deeply anesthetized and transcardiacally perfused with 50 mL heparinized PBS and 50 mL of 4 % PFA in PBS. Brains were removed and post-fixed for 2 h in 4 % PFA following cryoprotection in 20 % sucrose. Serial frozen coronal sections (thickness 7 μm) were cut by a cryostat (Leica Biosystems, IL, USA) and stored at −20 °C for immunostaining.

### Immunofluorescence

Cerebellar slices were fixed with 4 % PFA for 1 h at room temperature and then transferred to 12-well plates for immunostaining. Brain sections were air dried and immunostaining was done as described [35] with modification. After washed three times with PBS, brain sections or fixed cerebellar slices were incubated in PBS containing 1 % BSA, 0.1 % Triton X-100 in PBS and normal donkey serum (NDS, 2 % for sections and 10 % for cerebellar slices) at 4 °C overnight. Incubation for 1 h at room temperature was done with primary antibodies: rabbit anti-AQP4 (1:200, Santa Cruz Biotechnology, Santa Cruz, CA, USA), mouse anti-GFAP (1:100, Millipore, Temecula, CA, USA), goat anti-myelin basic protein (MBP) (1:200, Santa Cruz Biotechnology), rabbit anti-neurofilament (1:400, Millipore), rabbit anti-Iba1 (1:500, Wako, Richmond, VA, USA), rat anti-CD45 (1:50, Pharmingen, BD Biosciences, Oxford, UK), mouse anti-CC1 (1:100, Calbiochem), rabbit anti-Olig2 (1:100, Millipore), or rabbit anti-C5b-9 neo (1:50, Santa Cruz Biotechnology). After three washes with PBS, the appropriate species-specific Alexa Fluor-conjugated secondary antibody (1:500, Invitrogen) was added and incubated for 1 h at room temperature. Nuclei were counterstained blue with DAPI (Life technologies, Eugene, OR, USA). Fluorescence was visualized on a Leica DM 4000 B microscope or a Nikon confocal D-ELIPSE C1 system. AQP4, GFAP, MBP and NF immunonegative areas were defined by hand and quantified using ImageJ (version 1.43 m, National Institutes of Health, USA; http://rsbweb.nih.gov/ij). Data are presented as percentage of immunofluorescence loss area normalized to total area of brain hemisphere (or whole cerebellar slice).

### Statistics

Data are presented as mean ± S.E. Statistical analysis was performed using Prism 5 GraphPad Software package (GraphPad Software, San Diego, CA, USA). The normality of the data was established by Bartlett’s test for equal variances and a one-way ANOVA with Newmann-Keuls post-hoc test to compare groups. Individual statistical tests are reported in text and figure legends. The significance levels were set at *p* < 0.05 (*) and *p* < 0.01 (**).

## Results

### Clobetasol promotes OPC maturation and remyelination in vitro

**Fig. 1:**
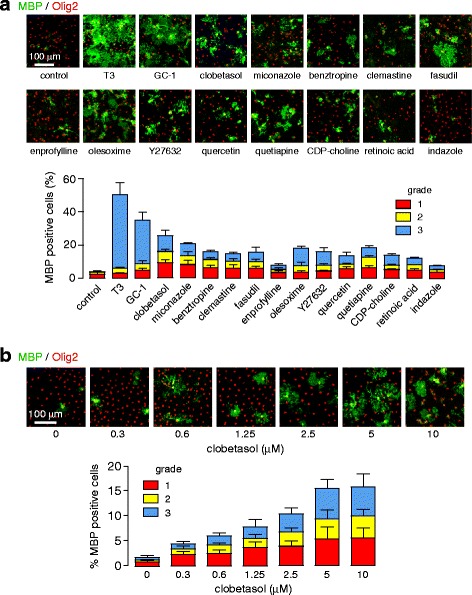
Evaluation of potential remyelinating drugs in primary cultures of oligodendrocyte precursor cells (OPCs). **a**. Representative immunofluorescence (Olig2, red; MBP, green) of OPC cultures treated for 6 days with vehicle (DMSO), T3 (30 nM) as positive control, and indicated drugs (GC-1 30 nM; benztropine, clemastine and fasudil 1 μM; others 5 μM) (*top*). Percentage of MBP-positive cells, with cells graded for degree of maturation (mean ± S.E., *n* = 18 fields in three wells, ** *P* < 0.01) (*bottom*). **b**. Clobetasol concentration-dependence study for OPC cultures treated for 6 days (*top*). Percentage of MBP-positive cells (mean ± S.E., *n* = 6 fields in three wells, ** *P* < 0.01) (*bottom*)

OPC maturation studies were done for the 14 potential remyelinating drugs listed in Table 1, together with negative control (vehicle alone) and positive control (T3). Representative fluorescence micrographs are shown in Fig. 1a (top), with immunostaining for MBP, a marker of myelin produced by mature oligodendrocytes, and Olig2, a marker of OPCs and mature oligodendrocytes. MBP-positive cells were graded into three levels (grade 1 to 3) according to their maturation level, as summarized in Fig. 1a (bottom). In vitro OPC maturation was confirmed for most of the drugs listed in Table 1, with strong maturation induced by GC-1, clobetasol and miconazole. Following preliminary evaluation showing greatest remyelinating efficacy in NMO models in cerebellar slices cultures and in mice, as described below, we focused attention on clobetasol, which was reported previously to enhance OPC differentiation and promote myelination in an in vitro phenotypic screen using stem cell-derived OPCs [24]. Figure 1b shows clobetasol concentration-dependence data, in which ~15 % of cells were MBP-positive after 6 days at 5 μM, which is the concentration used below in cerebellar slice cultures.

### Clobetasol promotes remyelination in AQP4-IgG treated cerebellar slice cultures

**Fig. 2:**
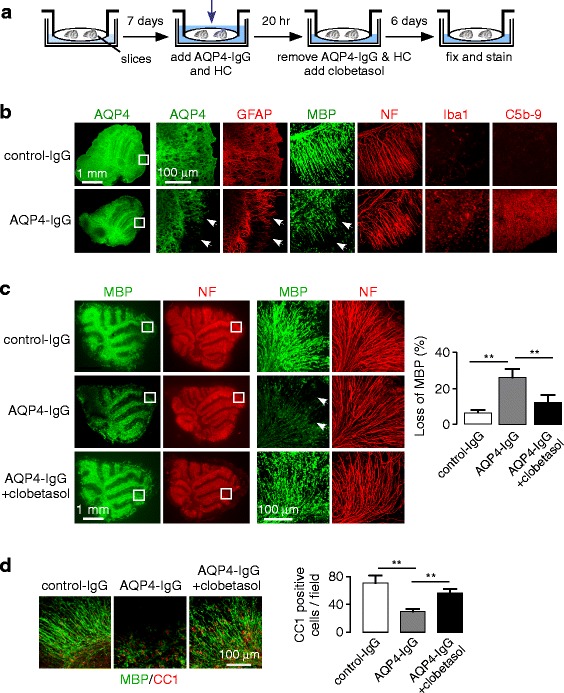
Clobetasol promotes remyelination in cerebellar slice cultures treated with AQP4-IgG and human complement. **a**. Mouse cerebellar slices were cultured for 7 days and then exposed for 20 h to AQP4-IgG (or control human IgG) and human complement (HC). Treatment with clobetasol (or vehicle control) was then started and slices were fixed for immunostaining 6 days later. **b**. Representative fluorescence images of control (non-NMO IgG) and AQP4-IgG treated cerebellar slices (both with human complement) showing immunostaining of astrocyte markers AQP4 and GFAP, myelin protein MBP, neuronal axon marker NF, microglial marker Iba-1, and activated complement C5b-9. **c**. AQP4, MBP and NF immunofluorescence in cerebellar slice cultures treated with AQP4-IgG and human complement, without or with clobetasol (*left*). Percentage loss of MBP immunofluorescence (mean ± S.E., 3 slices, ** *P* < 0.01) (*right*). **d**. High magnification confocal microscopy showing immunofluorescence of MBP and CC1 overlayed (*left*), with quantitation of CC1-positive cells per field (*right*)

To investigate whether clobetasol promotes remyelination in an ex vivo model of NMO, a previously described NMO slice culture model [48] was modified to enable studies of remyelination (Fig. 2a). As done in prior remyelination studies in a lysolecithin-induced demyelination model [24], here we used mouse cerebellar slice cultures in which long axonal segments are preserved and easily visualized. Culture conditions and the concentrations of AQP4-IgG and human complement were selected to give robust NMO pathology, with demyelination but minimal axonal injury (Fig. 2b). Cerebellar slices exposed to AQP4-IgG and human complement showed astrocyte cytotoxicity as seen by loss of AQP4 and GFAP immunofluorescence, inflammation as seen by Iba1 immunofluorescence, deposition of activated complement as seen by C5b-9 immunostaining, and myelin loss with axonal preservation as seen by MBP and NF immunofluorescence. These changes were not observed in cerebellar slices treated with control-IgG and human complement (Fig. 2b), or in slices exposed to AQP4-IgG without complement, or in slices from AQP4 knockout mice exposed to AQP4-IgG and human complement (data not shown).

For clobetasol experiments, we first confirmed in preliminary studies that 5 μM clobetasol promoted remyelination at 6 days following 20 h incubation with 0.5 mg/ml lysolecithin (data not shown), in agreement with prior published results [24]. As diagrammed in Fig. 2a, cerebellar slices were exposed to AQP4-IgG (or control human IgG) and 5 % human complement for 20 h, and then cultured for 6 days with clobetasol (or vehicle control). Fluorescence micrographs in Fig. 2c (left) showed significantly less myelin loss in the clobetasol-treated slices compared with control slices, as scored quantitatively in Fig. 2c (right). The number of mature oligodendrocytes was significantly greater in the clobetasol-treated slices, as seen by increased CC1 and Olig2 immunofluorescence (Fig. 2d). Clobetasol thus promotes oligodendrocyte maturation and remyelination in cerebellar slice cultures in which demyelination is produced by AQP4-IgG and complement.

### Clobetasol reduces myelin loss in a passive-transfer mouse model of NMO

**Fig. 3:**
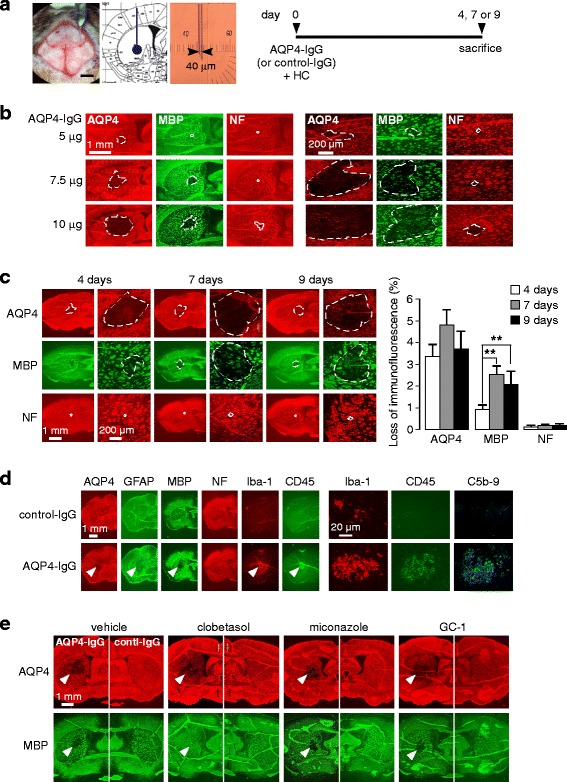
Mouse model of NMO for remyelination studies. **a**. Mice were infused with AQP4-IgG (or control IgG) containing human complement at day 0 using 40-μm tip diameter glass pipette and sacrificed at days 4, 7 or 9. Photographs show infusion (*left*), coordinates in striatum (*middle*) and glass pipette (*right*). **b**. Dose-determination study with indicated amounts of AQP4-IgG (each containing 30 % human complement) in a 3 μl total volume, showing AQP4, MBP and NF immunofluorescence at low (*left*) and high (*right*) magnifications. **c**. Time course of AQP4, MBP and NF immunofluorescence following intracerebral injection of 7.5 μg AQP4-IgG and 30 % human complement (*left*), with quantitative data (mean ± S.E., 6 mice, ** *P* < 0.01) (right). **d**. Brain sections from C were immunostained for indicated markers. **e**. Preliminary evaluation of potential remyelinating drugs (clobetasol 2 mg/kg/day, miconazole 10 mg/kg/day, GC-1 2 mg/kg/day). Mice were administered 7.5 μg AQP4-IgG or control-IgG and 30 % human complement at day 0 and treated with drugs (or vehicle) from days 1 to 9

A previously established mouse model of NMO produced by intracerebral injection of AQP4-IgG and human complement was modified for remyelination studies by optimizing concentrations, volumes, injection coordinates and measurement times in order to produce robust NMO pathology with demyelination but with minimal axonal injury. As diagrammed in Fig. 3a, mice were infused with 3 μl of a solution containing 7.5 μg AQP4-IgG (or control IgG) and 30 % human complement at coordinates 0.5 mm anterior and 2 mm lateral from the bregma. In an initial dose-finding study, different concentrations of AQP4-IgG and complement were tested. As seen in Fig. 3b, a low amount (5 μg) of AQP4-IgG produced little pathology, whereas a high amount (10 μg) produced marked pathology with loss of AQP4 and MBP immunofluorescence, with axonal damage and loss of NF immunofluorescence. An intermediate amount of AQP4-IgG (7.5 μg) and human complement (30 %) produced robust NMO pathology with myelin loss but minimal axonal damage.

A time course study was done to characterize the model. Mice were sacrificed at 4, 7 and 9 days after intracerebral injection of 3 μl of PBS containing 7.5 μg AQP4-IgG and 30 % human complement. Figure 3c shows astrocyte damage with loss of AQP4 and GFAP immunofluorescence, and demyelination at 4, 7 and 9 days, with little loss of NF immunofluorescence. There was relatively less demyelination at 4 days. By 9 days there was some evidence of reactive gliosis as seen by AQP4 staining. In the contralateral hemisphere (injected with control IgG), no pathology was seen except very near the needle tract. At high magnification at 9 days, there was inflammation and deposition of activated complement in the AQP4-IgG-treated mice, as seen by Iba1, CD45 and C5b-9 immunofluoresence (Fig. 3d). In control studies, these changes were not seen in mice injected with AQP4-IgG alone or control (non-NMO-IgG) IgG and human complement, or in AQP4 knockout mice injected with AQP4-IgG and human complement (data not shown), in agreement with prior studies done using similar mouse models [35, 36]. Figure 3e shows exemplary data using the model for 3 candidate remyelinating drugs, clobetasol, miconazole and GC-1, which was in part the basis for the focus on clobetasol. Less myelin loss was seen in clobetasol-treated mouse brain comparing with miconazole and GC-1.

**Fig. 4:**
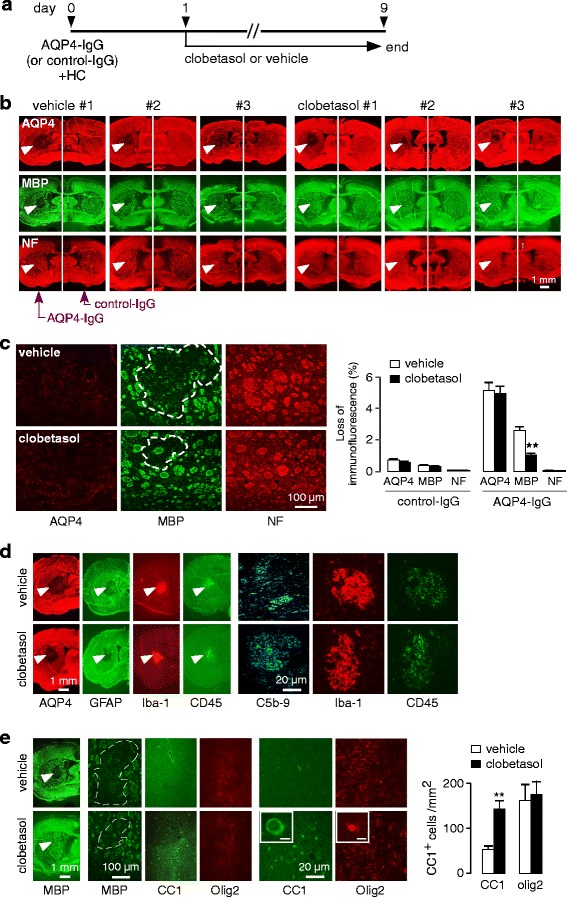
Clobetasol reduces myelin loss in a mouse model of NMO produced by intracerebral injection of AQP4-IgG and human complement. **a**. Protocol showing AQP4-IgG (or control IgG) injected at day 0, with clobetasol (or vehicle) treatment given from days 1 to 9. **b**. Gallery of micrographs showing AQP4, MBP and NF immunofluorescence for three clobetasol- and three vehicle-treated mice. **c**. Representative immunofluorescence with high magnification of AQP4, MBP and NF (*left*); summary of area of loss of immunofluorescence (normalized to total area of brain hemisphere) (mean ± S.E., 6 mice per group, ** *P* < 0.01) (*right*). **d**. Immunofluorescence for indicated markers. **e**. High-magnification confocal microscopy showing MBP, CC1 and Olig2 immunofluorescence (*left*), with quantitative data of CC1-positive cells per mm^2^ (mean ± S.E., 3 mice per group, six fields per section, ** *P* < 0.01) (*right*)

To test the efficacy of clobetasol to promote remyelination in NMO in vivo, mice were treated with clobetasol from days 1 to 9 (Fig. 4a). The dose of clobetasol (2 mg/kg/day, i.p.) was the same as that reported to promote remyelination in lysolecithin and EAE mouse models [24]. Figure 4b shows representative low magnification immunofluorescence from three control (vehicle) and three clobetasol-treated mice, each of which received 3 μl of PBS containing 7.5 μg AQP4-IgG or control-IgG and 30 % human complement, with high magnification confocal micrographs shown in Fig. 4c (left). There was reduced myelin loss (MBP staining) in the clobetasol-treated than control mice, with minimal axonal damage (NF staining) and similar astrocyte damage (AQP4 immunofluorescence). The loss of AQP4, MBP and NF immunofluorescence is summarized in Fig. 4c (right). Figure 4d shows similar inflammation (Iba-1 and CD45 immunofluorescence) and complement activation (C5b-9 immunofluorescence) in the clobetasol- and vehicle-treated mice. A significant increase in the number of mature, differentiated oligodendrocytes was seen in and around lesions in the clobetasol-treated mice, though the numbers of oligodendrocyte precursor cells was similar (Fig. 4e).

### Clobetasol promotes remyelination in NMO

**Fig. 5:**
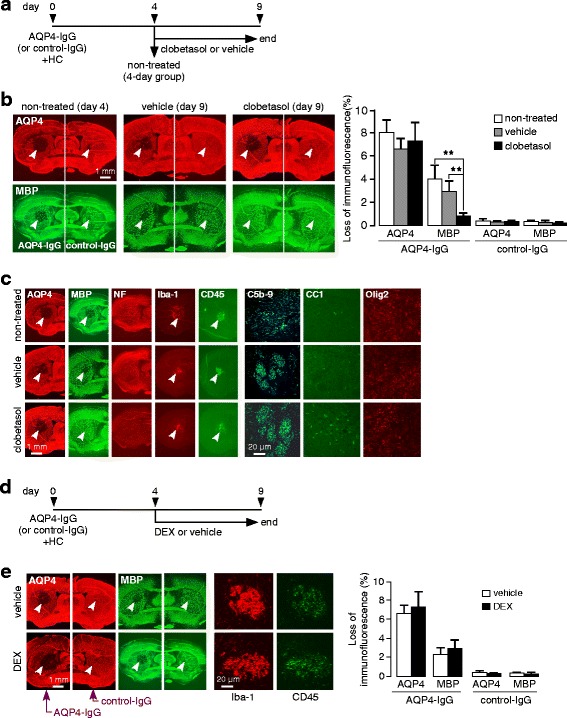
Clobetasol promotes remyelination in NMO. **a**. Mice were treated with clobetasol or vehicle starting on day 4 and sacrificed at day 9. One group of mice were sacrificed at day 4 without treatment (‘non-treated’ mice). **b**. Immunofluorescence of AQP4 and MBP from non-treated (day 4), vehicle-treated (day 9) and clobetasol-treated (day 9) mice; arrows point to site of infusion (*left*). Areas of loss of AQP4 and MBP immunofluorescence normalized with brain hemisphere area (mean ± S.E., 3 mice per group, ** *P* < 0.01) (*right*). **c**. Immunofluorescence for indicated markers. **d**. Mice were treated with dexamethasone (DEX) or vehicle starting on day 4 and sacrificed at day 9. **e**. Representative immunofluorescence (*left*) with quantitative data for loss of AQP4 and MBP immunofluorescence (mean ± S.E., 3 mice per group, differences not significant) (*right*)

The above data suggest that clobetasol promotes remyelination rather than inhibiting inflammation or reducing initial astrocyte damage, as clobetasol increased the number of mature oligodendrocytes in NMO lesions without affecting the loss of AQP4 immunofluorescence or inflammation. As further evidence that clobetasol promotes remyelination in our model, mice were treated with clobetasol (or vehicle control) starting 4 days after intracerebral administration of AQP4-IgG and human complement, a time at which demyelination and the primary inflammatory response has occurred (Fig. 5a). Mice sacrificed on day 4 showed loss of AQP4 and MBP immunofluorescence; on day 9 there was some remyelination in the vehicle-treated mice, which was much greater in the clobetasol-treated mice (Fig. 5b). Immunofluorescence showed similar astrocyte damage (AQP4) and inflammatory response (Iba-1 and CD45), with an increased number of CC1-positive mature oligodendrocytes (Fig. 5c).

To further investigate whether the corticosteroid action of clobetasol might be involved in reducing myelin loss in our model, mice were treated with high-dose dexamethasone (3 mg/kg/day) at day 4 and sacrificed at day 9 (Fig. 5d). Immunofluorescence showed similar loss of AQP4 and MBP in the control and dexamethasone-treated mice, and a similar inflammatory response (Fig. 5e).

## Discussion

Remyelination has received considerable attention as a potential therapeutic strategy in multiple sclerosis, with several drugs in pre-clinical development or clinical trials, including anti-Lingo-1 antibody, antagonists of M1 and/or M3 muscarinic receptors (benztropine), antihistamine and anticholinergic drugs (clemastine), and mitochondrial pore modulators (TRO19622) [8, 20, 50]. In addition, screens of approved and investigational drugs with oligodendrocyte precursor cells have yielded additional candidate drugs for potential repurposing in demyelinating disorders [24], some of which are listed in Table 1. The study here was done to investigate the appropriateness of remyelination for NMO therapy. Because NMO pathogenesis involves primary astrocyte cytotoxicity and an inflammatory environment with blood–brain barrier disruption, it was not clear a priori whether drug-induced OPC maturation could promote remyelination in NMO. Our results support this possibility, as clobetasol produced significant remyelination in a mouse model of NMO produced by passive-transfer of AQP4-IgG. However, it is difficult to predict with confidence whether this conclusion from a mouse model will translate to human NMO, though recent findings showing similar early axonal loss in NMO and multiple sclerosis [3] supports the possibility of a remyelination approach in NMO, at least as an early intervention during a disease exacerbation before gliosis and axonal injury occurs.

The in vivo mouse model of NMO chosen for the studies here involved a single, stereotaxic injection of a recombinant monoclonal AQP4-IgG and human complement into brain under conditions that produce robust, reproducible NMO pathology with loss of AQP4 and GFAP immunoreactivity, inflammation, complement deposition, and, most importantly, demyelination with minimal axonal loss. The single injection model used here, as adapted from the original description [36] and follow-on applications [30, 32, 35], was modified with regard to injection details and amounts of AQP4-IgG and complement to give demyelination with minimal axonal injury. A pulled glass pipette with 40-μm tip diameter minimized traumatic brain tissue injury, as did the minimal volume of injected fluid and the slow infusion rate and pipette withdrawal. We did not use continuous AQP4-IgG infusion models, as described [49], for technical simplicity and to avoid potential pathology caused by chronic needle placement. We did not use rat models, as described [1], because the pharmacology and efficacy of the drugs tested had been established in mice, as well as practical considerations in creating the model in a sufficient number of animals to yield statistically significant differences. It is acknowledged, however, that available animal models of NMO are imperfect, as NMO pathology requires invasive, passive-transfer of AQP4-IgG rather than spontaneous autoimmunity, and because of differences between rodents and humans in astrocyte:neuron ratios and their biology.

Our approach was to evaluate drug candidates that were reported to induce differentiation and/or proliferation of OPC cultures, and promote remyelination in cerebellar slices and mice following AQP4-IgG-induced demyelination. Following initial evaluation in pure OPC cultures and cerebellar slices, the most efficacious compounds were screened in the mouse model of NMO, from which clobetasol was selected for full analysis. Because the study here was done primarily for proof of concept we were not overly concerned with selection of the very best molecule for clinical development. Though we think it unlikely, it is possible that an NMO-specific remyelination drug screen might yield alternative, more efficacious compounds. It is not practical, however, to screen thousands of molecules for efficacy in a model of NMO demyelination.

Clobetasol was discovered as a potential remyelinating agents in a screen of 727 drugs in an in vitro phenotypic assay using mouse epiblast stem cell (EpiSC)-derived OPCs [24]. Another screen of 1200 FDA-approved drugs in a mouse immortalized oligodendrocyte cell line identified clobetasol as one of the top ranking compounds in promoting myelin basic protein expression [34]. Clobetasol is a FDA-approved topical corticosteroid used clinically for the treatment of various skin disorders including eczema, psoriasis, alopecia areata, vitiligo, lichen sclerosus and lichen planus. Unlike benztropine and clemastine, clobetasol does not inhibit muscarinic receptor subtypes (M1-M5), nor does it inhibit various kinase isoforms [24]. As a corticosteroid, clobetasol modulates glucocorticoid receptor signaling in cytoplasm. Clobetasol has been shown to promote Schwann-cell-mediated myelination in the peripheral nervous system [23] and functions as a smoothened (Smo) agonist [43, 44] that activates Hedgehog signaling and stimulates OPC proliferation and differentiation [10, 27, 34]. Clobetasol is currently approved only for topical administration in humans; however, recent pharmacokinetics data in rodents [24] showed effective blood–brain barrier penetration following systemic administration. Several lines of evidence suggest that the remyelinating efficacy of clobetasol in our model does not involve a glucocorticoid anti-inflammatory effect, but rather an action on OPCs, as: (i) clobetasol induced OPC maturation in pure OPC cultures; (ii) clobetasol promoted remyelination in cerebellar slices; (iii) clobetasol was effective when given after primary astrocyte injury, inflammation and demyelination; and (iv) high-dose dexamethasone did not promote remyelination. Though dexamethasone has anti-inflammatory actions, it did not have effect here probably because the acute inflammation in our model is largely resolved by 4 days. Like clobetasol, dexamethasone has effects on Smo and Hedgehog signaling pathways [25, 43, 46], though there is no literature on dexamethasone action on OPC maturation.

Though our results provide proof of concept for the potential utility of remyelinating drugs in NMO, several caveats are noted in extrapolating the data here to predicting the efficacy of remyelinating drugs in human NMO. As mentioned above, there are differences in the proportions of astrocytes vs. neurons in rodents and humans, and there may be differences in the biology of oligodendrocyte maturation and interaction with astrocytes and microvascular endothelia. There may be differences between mice and humans in drug pharmacokinetics and penetration into the central nervous system. With regard to our mouse model of NMO produced by passive, intracerebral transfer of AQP4-IgG, though it recapitulates the major pathological features of human NMO, it is far from the ideal, not yet realized model of NMO in which spontaneous AQP4 autoimmunity produces NMO pathology in spinal cord, optic nerve and brain. Finally, we note that remyelination requires intact axons and neuronal viability, which may be heterogeneous in timing and extent in human NMO.

## Conclusion

In summary, our results provide evidence for remyelinating therapy in NMO, which might be most effective when administered early during a disease exacerbation. As remyelination requires intact axons, therapeutics promoting remyelination may be of limited benefit when administered late in the course of NMO. Because of their distinct mechanisms of action and targets, remyelination therapeutics may be efficacious in combination with therapeutics used currently in NMO, as well as therapeutics in the development pipeline. For example, AQP4-IgG targeted approaches, plasma exchange and immunosuppressants target upstream disease-initiating events, inflammation and demyelination, while remyelinating drugs would protect against neuronal injury and reduce the cumulative neurological deficit. Our data support clinical testing of remyelinating drugs in NMO.
